# Evaluation of a Mindfulness-Based Intervention With and Without Virtual Reality Dialectical Behavior Therapy^®^ Mindfulness Skills Training for the Treatment of Generalized Anxiety Disorder in Primary Care: A Pilot Study

**DOI:** 10.3389/fpsyg.2019.00055

**Published:** 2019-01-28

**Authors:** María V. Navarro-Haro, Marta Modrego-Alarcón, Hunter G. Hoffman, Alba López-Montoyo, Mayte Navarro-Gil, Jesús Montero-Marin, Azucena García-Palacios, Luis Borao, Javier García-Campayo

**Affiliations:** ^1^Personality Disorders Unit, General University Hospital of Catalonia, University of Barcelona, Barcelona, Spain; ^2^Instituto de Investigación Sanitaria Aragón, Zaragoza, Spain; ^3^Virtual Reality Research Center at the Human Photonics Lab, Mechanical Engineering, University of Washington, Seattle, WA, United States; ^4^Edificio Investigación II, Universitat Jaume I, Castellón de la Plana, Spain; ^5^Primary Care Prevention and Health Promotion Network, RedIAPP, Zaragoza, Spain; ^6^Hospital Universitario Miguel Servet, Zaragoza, Spain

**Keywords:** virtual reality, mindfulness, generalized anxiety disorder, virtual reality mindfulness, dialectical behavior therapy

## Abstract

Generalized Anxiety Disorder (GAD) is a very prevalent disorder in primary care (PC). Most patients with GAD never seek treatment, and those who do seek treatment often drop out before completing treatment. Although it is an understudied treatment, Mindfulness-Based Interventions (MBIs) indicate preliminary efficacy for the treatment of GAD symptoms, but many patients with GAD present other associated symptoms (e.g., attention deficits) that complicate the treatment. Virtual Reality DBT^®^ Mindfulness Skills learning has recently been developed to make learning mindfulness easier for patients with emotion dysregulation who have trouble concentrating. Virtual Reality (VR) might serve as a visual guide for practicing mindfulness as it gives patients the illusion of “being there” in the 3D computer generated world. The main goal of this study was to evaluate the effect of two MBIs (a MBI in a group setting alone and the same MBI plus 10 min VR DBT^®^ Mindfulness skills training) to reduce GAD symptoms. A secondary aim was to explore the effect in depression, emotion regulation, mindfulness, and interoceptive awareness. Other exploratory aims regarding the use of VR DBT^®^ Mindfulness skills were also carried out. The sample was composed of 42 patients (roughly half in each group) with GAD attending PC visits. After treatment, both groups of patients showed significant improvements in General Anxiety Disorder measured by the GAD-7 using mixed regression models [MBI alone (*B* = -5.70; *p* < 0.001; *d* = -1.36), MBI+VR DBT^®^ Mindfulness skills (*B* = -4.38; *p* < 0.001; *d* = -1.33)]. Both groups also showed significant improvements in anxiety, depression, difficulties of emotion regulation and several aspects of mindfulness and interoceptive awareness. Patients in the group that received additional 10 min VR DBT Mindfulness Skills training were significantly more adherent to the treatment than those receiving only standard MBI (100% completion rate in MBI + VR vs. 70% completion rate in MBI alone; *Fisher* = 0.020). Although randomized controlled studies with larger samples are needed, this pilot study shows preliminary effectiveness of MBI to treat GAD, and preliminary evidence that adjunctive VR DBT^®^ Mindfulness Skills may reduce dropouts.

## Introduction

Generalized anxiety disorder (GAD) is defined as “the presence of excessive anxiety and worry about a variety of topics, events, or activities. Worry occurs for at least 6 month and is clearly excessive” (DSM-5, American Psychiatric Association, 2013). Excessive worry means worrying even when there is no threat or worrying in a manner that is disproportionate to the actual risk and spending a large amount of time worrying about something. The anxiety and worry occur normally together with other physical or cognitive symptoms such as being easily fatigued, muscle tension, difficulty concentrating, restlessness, irritability, and disturbed sleep. People diagnosed with GAD do not always identify their worries as irrational but they report distress due to constant worry and have trouble controlling their anxiety. GAD symptoms are related to impairment in social, occupational, or other important areas of functioning (American Psychiatric Association, 2013). Unlike other anxiety disorders usually associated with specific stimuli or situations, GAD is characterized by a constant and unspecific anxiety, involving a process of interacting systems (attentional, conceptual, imaginal, physiological, affective, and behavioral) that unfolds over time in continual response to a constantly changing environment (Gorini and Riva, 2008). Due to its high interference with the patients everyday life, GAD is one of the most frequently observed problems in primary care (PC) and its prevalence is around 10% of the people with a mental disorder visiting PC (Lieb et al., 2005).

Allgulander (2006) estimated that 2/3rds of the patients that suffer of GAD never receive treatment for GAD (for a number of reasons, including perceived stigma, Hoge et al., 2013). The complete symptoms remission after 5 years of starting clinical treatment for the disorder is achieved only in 18–35%. Furthermore, GAD is highly associated with comorbid mental disorders; major depressive disorder is the most frequent (Tyrer and Baldwin, 2006), making treatment more challenging. More effective treatments for GAD are needed.

Patients with GAD have shown significantly lower levels of mindfulness and higher emotional dysregulation than a non-anxious control group, suggesting that mindfulness training could help GAD patients (Roemer et al., 2009, see also Hoge et al., 2013). Worrying uses up attentional resources, reducing the patient’s ability to pay attention during therapy (American Psychiatric Association, 2013). Therefore, treatments, such as those focused on mindfulness, that target attention intentionally and reinforce the sustained attention are needed. Cognitive Behavior Therapy (CBT) has shown some evidence – although no more than relaxation therapy (Montero-Marin et al., 2017) – for reducing GAD symptoms, but high rates of drop-out during CBT groups for GAD have been found (Gale and Davidson, 2007; Hunot et al., 2007). Recent Mindfulness-based interventions (MBIs) have shown promise for the treatment of depression, anxiety and adjustment disorders (e.g., Sundquist et al., 2017). Pre-post studies evaluating mindfulness-based interventions with small samples of patients suffering from GAD have shown significant improvements in pathological worry (Dahlin et al., 2016), stress and quality of life (Craigie et al., 2008), as well as reductions in anxiety and depressive symptoms (Evans et al., 2008). To our knowledge, there is only one randomized controlled trial that has evaluated the effect of mindfulness in the treatment of people diagnosed with GAD. Mindfulness Based Stress Reduction (MBSR) program (consisted of eight week) was compared to a Stress Management Education program based on attention control. Using a laboratory stress paradigm to measure anxiety and stress (i.e., measured only a subset of GAD symptoms), participants receiving MBSR showed greater reductions in anxiety symptoms, stress reactivity and coping than the control group (Hoge et al., 2013). MBIs conducted in a group setting have also been considered preliminary effective for GAD as co-adjunct of psychopharmacology in a recent meta-analysis (Hodann-Caudevilla and Serrano-Pintado, 2016).

One challenge to using traditional MBIs to treat GAD is that many GAD patients have attention deficits because worrying is distracting, and depression reduces attentional resources (American Psychiatric Association, 2013). Gorini and Riva (2008) suggested that the small number of studies with Virtual Reality (VR) for GAD is related to the difficulty to develop standardized VR scenarios that capture the numerous, varying, individualized worries of patients suffering from GAD. To deal with this problem, VR could serve as a tool to enhance MBIs that target important areas associated with GAD such as attention and awareness (Maples-Keller et al., 2017). Virtual Reality DBT^®^ Mindfulness Skills training has recently been developed to make learning mindfulness easier for patients with emotion dysregulation who have attention deficits or reduced attentional resources (Navarro-Haro et al., 2016; Gomez et al., 2017; Flores et al., 2018). Patients “go into” a 3D computer generated world, which blocks out distractions from the real world. Immersive VR is designed to allow the computer user a sense of presence, defined as the “illusion of going inside the computer-generated world, as if it is a place they are visiting” (Slater et al., 1994). VR gives patients the illusion of “being there” in the 3D computer generated world, and the essence of mindfulness is to “be here” in the present moment. During VR DBT^®^ Mindfulness Skills training, patients are encouraged to “be here now” in the computer-generated world.

The current study further explores the use of mindfulness-based therapy for treating GAD, and this is the first controlled clinical study to use Virtual Reality DBT^®^ Mindfulness Skills training as an adjunct. We reason that the illusion of “presence” in virtual reality might help patients practice mindfulness, and that VR may make mindfulness more interesting. The main objective of the present study was to evaluate the effect of a MBI in a group setting (alone and plus VR DBT^®^ mindfulness skills training) to reduce GAD symptoms. The secondary aim was to evaluate the effect of both interventions in other problems associated with GAD (e.g., depression, emotion dysregulation, interoceptive awareness and levels of mindfulness). Other exploratory aims specifically directed to the MBI + VR DBT^®^ Mindfulness Skills training group were: to assess possible effects of this group – and other possible sociodemographic and psychological predictors – in treatment adherence; to evaluate possible short-term improvements in emotional state through each of the MBI + VR sessions; and to assess sense of presence along the treatment with MBI + VR.

Our primary hypothesis was that participants receiving both the MBI alone and the MBI + VR DBT^®^ Mindfulness Skills training would show significant decreases in GAD symptoms. As exploratory hypotheses, we expected that both groups would be efficacious for the improvement of the other problems associated with GAD; the group receiving MBI + VR DBT^®^ Mindfulness Skills training would be more adherent to the treatment than the MBI alone. Participants of the MBI + VR DBT^®^ Mindfulness Skills training group would improve their emotional state in each of the VR sessions and would increase sense of presence during the treatment.

## Materials and Methods

### Participants

Participants were recruited from three public centers (PC) of Zaragoza, Spain. To contact participants the research team informed the PC doctors of these three centers about the aims of the study. Doctors were asked to select patients with anxiety symptoms and indications of GAD. Doctors then derived the possible candidates for a psychological assessment with an assessor in psychology that was part of the research team. The assessor carried out a *screening* to evaluate whether participants met inclusion/exclusion criteria. Inclusion criteria were: (a) diagnosis of GAD by DSM using the Mini International Neuropsychiatric Interview; (b) being between 18 and 65 years old; (c) speaking and understanding of Spanish. Exclusion criteria were: (a) being pregnant; (b) a diagnosis of Obsessive Compulsive Disorder or other Anxiety Disorder by DSM-5 (American Psychiatric Association, 2013); (c) receiving other psychological treatment during the intervention. In addition, an increment of pharmacological medication during the intervention was also an exclusion criterion. Participants meeting the criteria were invited to participate the present study. Based on previous studies (Craigie et al., 2008; Evans et al., 2008), it was assumed that a large effect size in the pre-post primary outcome of GAD-7 would be obtained. Using a 95% confidence interval in a bilateral test and a statistical power of 80%, considering a dropout rate of roughly 25%, we estimated around 20 subjects in each group would be needed to test their respective pre-post differences, which coincides with the sample size used in the study of Craigie et al. (2008).

**FIGURE 1:**
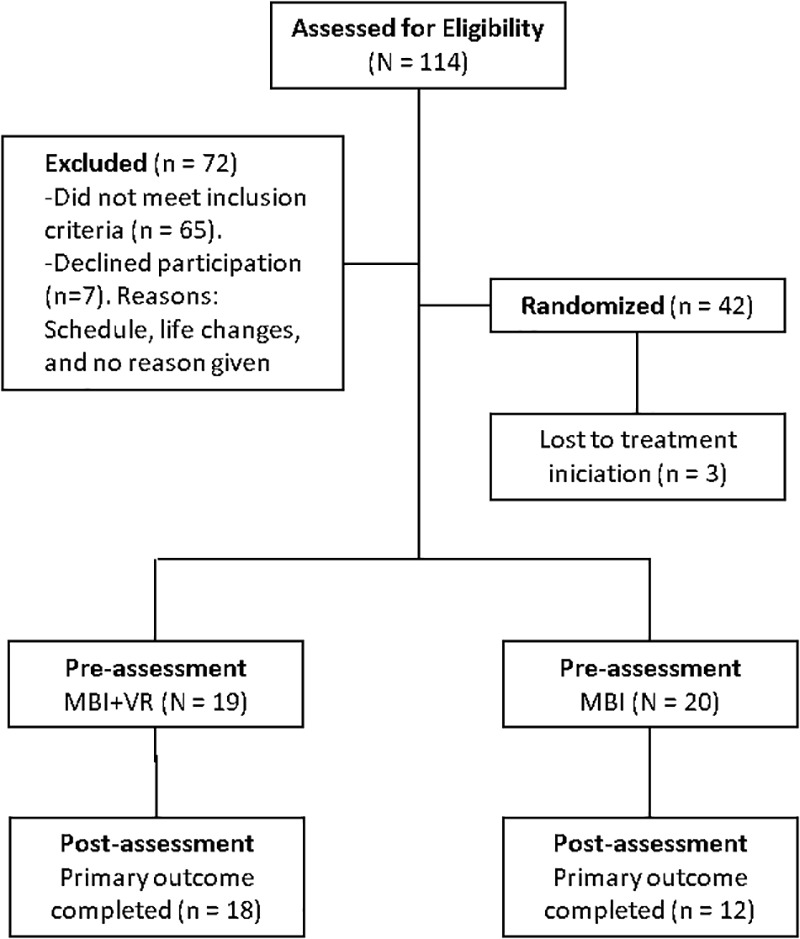
Flow chart.

Figure 1 presents the participant’s study flow. The total sampling universe of the study was composed of 114 patients. Of those, 72 were excluded and 42 participants were eligible to participate in the study. Three out of forty two participants were lost before treatment initiation, and therefore the final sample was composed by thirty nine patients. They were randomly assigned either to the MBI group (*n* = 20) or to the MBI + VR group (*n* = 19). Participants were middle aged, and most of them were women. Around half of participants were with a partner, had secondary studies, and were employed and taken medication. See Table 1 for a detailed description of demographic information for the total sample and by group.

**Table 1:** Baseline characteristics of participants by group.

Variables	Total (*n* = 39)	MBI (*n* = 20)	MBI + VR (*n* = 19)	*p*
**Socio-demographic**				
Age, mean (SD)	45.23 (11.23)	45.40 (13.74)	45.05 (8.17)	0.924
Gender (females), *n* (%)	30 (76.9)	15 (75.0)	15 (78.9)	0.535
Married/partner, *n* (%)	22 (56.4)	10 (50.0)	12 (63.2)	0.408
**Studies, *n* (%)**				
Primary	6 (15.4)	3 (15.0)	3 (15.8)	0.909
Secondary	18 (46.2)	10 (50.0)	8 (44.4)	
University	15 (38.5)	7 (35.0)	8 (42.1)	
**Employment, *n* (%)**				
Employed	19 (48.7)	9 (45.0)	10 (52.6)	0.702
No-employed	11 (28.2)	7 (35.0)	4 (21.1)	
Sick leave/retired	9 (23.1)	4 (20.0)	5 (26.3)	
Medication (yes), *n* (%)	26 (66.7)	12 (60.0)	14 (53.8)	0.365
**Psychological**				
GAD-7, mean (SD)	14.44 (4.24)	14.80 (3.94)	14.05 (4.61)	0.589
**HADS, mean (SD)**				
Anxiety	13.31 (3.20)	13.55 (2.09)	13.05 (4.10)	0.634
Depression	9.28 (3.28)	9.90 (2.79)	8.63 (3.70)	0.233
**FFMQ, mean (SD)**				
Observing	26.21 (5.23)	24.20 (4.88)	28.32 (4.83)	0.740
Describing	24.72 (6.34)	23.45 (7.16)	26.05 (5.20)	0.387
Acting with awareness	20.44 (4.93)	20.94 (4.36)	19.94 (5.53)	0.528
Non-judging	16.13 (6.78)	15.90 (6.94)	16.37 (6.80)	0.862
Non-reactivity	19.51 (3.96)	18.80 (3.90)	20.26 (3.98)	0.928
**DERS, mean (SD)**				
Inattention	10.72 (3.97)	11.88 (3.94)	9.68 (3.89)	0.098
Confusion	9.72 (3.43)	10.47 (4.20)	9.05 (2.48)	0.220
Non-acceptance	21.76 (8.28)	23.19 (7.72)	20.50 (8.77)	0.353
Interference	13.72 (3.49)	14.29 (3.53)	13.21 (3.47)	0.360
Impulse	24.62 (8.13)	26.06 (8.39)	23.33 (7.90)	0.336
**MAIA, mean (SD)**				
Noticing	3.99 (0.53)	3.84 (0.61)	4.15 (0.39)	0.068
Distracting	2.00 (1.00)	1.87 (1.13)	2.15 (0.85)	0.394
Not-worrying	1.96 (1.11)	2.12 (1.19)	1.80 (1.02)	0.382
Attention-regulation	2.77 (1.11)	2.52 (1.13)	3.06 (1.03)	0.143
Emotional-awareness	4.41 (1.39)	4.46 (1.86)	4.34 (0.59)	0.802
Self-regulation	2.30 (1.05)	2.13 (0.99)	2.49 (1.12)	0.297
Body-listening	2.15 (1.43)	1.67 (1.37)	2.69 (1.33)	0.026
Trusting	2.48 (1.52)	2.10 (1.72)	2.91 (1.17)	0.104


*MBI = mindfulness-based intervention; MBI + VR = mindfulness-based intervention + virtual reality; *p* = *p*-value as a result of χ^*2*^ (or Fisher) test for categorical variables, and *t*-test for continuous variables.*

### Procedure

Participants were evaluated at the Primary Healthcare public center of Arrabal (Zaragoza, Spain). Participants who met inclusion criteria were informed about the goal of the study, and if they agreed to participate, signed the written informed consent. Participants were randomly assigned to receive one of the two conditions before starting the treatment: (1) Mindfulness-based intervention in a group setting for GAD without VR (MBI); or (2) MBI for GAD plus 10 minute VR DBT^®^ Mindfulness skills training (MBI+VR DBT^®^) before or after each MBI session. The whole sample completed a battery of measures a week before the treatment (pre-treatment), and after treatment in the last session of the intervention (post-treatment). In addition, the MBI + VR DBT^®^ group, filled out additional measures before and after each VR DBT^®^ Mindfulness Skills learning session. The survey was administered by a psychologist research assistant, who was blinded to treatment conditions, and who answered any questions patients had regarding the questionnaires.

Participants randomly assigned to the MBI condition received seven MBI group sessions (one per week with duration of 90 min per session). The sample allocated in the MBI + VR DBT condition received six MBI group sessions (one per week with duration of 90 min per session, with no VR) and six individual additional sessions (15 min per session: 10 min of mindfulness exercises and 5 min to complete the study measures) of VR DBT^®^ Mindfulness Skills training per week. The time of intervention was controlled by matching the two conditions with the same time, a total of 630 min of intervention per group, and therefore, the last MBI session of the MBI + VR DBT^®^ group was eliminated to match the time. Nonetheless, participants had a close-up session in the last VR session. Participants in both conditions attended the MBI together. Sessions of VR mindfulness were conducted before or after the group individually. The VR practice was received individually due to current technology requirements (e.g., having to use Oculus Rift and a computer) that currently impeded to do it in group. Instructions to using VR as well as questions about how to complete the specific VR measures were facilitated by a distinct psychologist research assistant of the team.

### Interventions

#### Mindfulness-Based Intervention Group (MBI)

The no-VR portion of the intervention is based on the mindfulness-based program developed by García-Campayo and Demarzo (2014). This program is composed of seven modules, one per week, with a duration of 90 min each one. In the first module, the mindfulness concept and its main characteristics are defined. Module 2 consists of teaching the practice of breathing meditation (e.g., focusing in physical sensations of the breathing), considered to be one of the formal practices of mindfulness. In the module three, body scan meditation (e.g., noticing sensations of each part of the body gradually), another formal practice, is explained. Module 4 teaches examples of different informal mindfulness practices (e.g., noticing the environment when we are walking), with the aim that participants practice mindfulness outside of therapy, in their everyday life. In the module 5, the concept of radical acceptance is introduced, and it is defined and explained as a way to deal with emotional distress. The goal is that the person realizes that there are situations that cannot be changed, and that trying to fight with those situations results in losing what it is meaningful, the person’s values. Module six aims to target the compassion concept from an oriental perspective, and differences between oriental and occidental perspectives are discussed. In the last module, the number seven, all the previous modules are reviewed, and the formed group is closured.

#### MBI Plus Adjunctive 10 min Virtual Reality Mindfulness Skills Training (MBI + VR DBT^®^)

**FIGURE 2:**
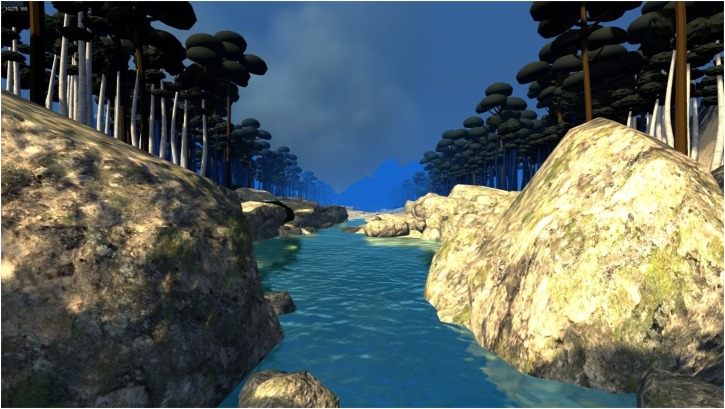
A screenshot of DBT VR *MindfulRiverWorld*. Image by bigenvironments.com, copyright Hunter Hoffman, UW, www.vrpain.com.

Participants in this condition received the first six sessions of the MBI group described above and six individual additional ten-minute sessions of VR DBT^®^ mindfulness skills training per week. During the VR sessions, participants were seated in a chair and wore a pair of Oculus Rift DK2 VR goggles with head mounted display, with head tracking. This allowed them to see the 3D computer generated river DBT^®^ VR *MindfulRiverWorld*^1^ with the help of a MSI GT Series GT72 Dominator Pro G-1252 Gaming Laptop 6th Generation Intel Core i7 6700HQ (2.60 GHz) 16 GB Memory 1 TB HDD 512 GB SSD NVIDIA GeForce GTX 980M 4 GB GDDR5 17.3” Windows 10 Home 64-Bit while listening to one of the DBT^®^ mindfulness skills practices using Bose Q25 headphones. The visuals of DBT^®^ VR *MindfulRiverWorld* (see Figure 2) were developed to give the participant the illusion of going inside the 3D computer generated world, where they floated slowly down a computer generated river in VR, with trees, boulders, and mountains (Navarro-Haro et al., 2016).

Participants listened to DBT^®^ mindfulness skills learning audios (copyrighted by Marsha Linehan) while floating down the river generated by VR. The three audios used during the VR DBT^®^ mindfulness skills training were adapted and translated into Spanish. Participants were randomly assigned to listen to one of three 10 minute DBT^®^ Skills Learning practices: “observing sounds”, “observing visuals”, or “wise mind” (pooled for analyses). The observing sounds audio was developed to learn how to focus their attention on noticing sounds, and repeatedly bringing attention back to sounds every time the mind wanders off (Linehan, 2002). The observing visuals audio teaches patients to observe just what they saw in the moment, to notice, not to let their attention get fixed upon anything, and to bring their attention back, if it became distracted. The wise mind (synthesis or integration of opposites: emotion mind and reasonable mind, Linehan, 1993, 2015) audio was adapted from a DBT^®^ mindfulness exercise named “Stone flake on the lake” (Linehan, 2015) to match the visuals of floating down the river, where the bottom of the river represents the inner wise mind, the ocean of wisdom (Navarro-Haro et al., 2016).

### Measures

#### Pre-intervention Assessments

##### Demographic information

Demographic information was collected with a questionnaire developed by our team. The demographics gathered information about age, gender, marital status (married or with partner vs. with no partner), educational level (primary, secondary, university), employment status (employed, no-employed, sick leave/retired), and whether they were taking medication (yes, no). This information was collected a week before the beginning of the intervention.

##### Mini international neuropsychiatric interview (MINI; Sheenan et al., 1998; Ferrando et al., 2000)

The MINI is a brief structured and diagnostic interview that explores 17 disorders based on DSM criteria. It can be used for clinicians after a brief training. This study only used the section dedicated to evaluate GAD. A score higher to five in this scale meant diagnosis of GAD. Participants with a score of five or higher were selected for the study. This measure has shown good inter-rater reliability for diagnosing GAD (Cohen, 1988; Hergueta et al., 1998). When the patient was referred by the doctors at primary care, this interview was administered by an assessor in psychology to evaluate whether participants met criteria for GAD diagnosis.

#### Pre-post Treatment Assessments

Pre-post assessments were completed by participants a week before the treatment (pre-treatment), and after treatment in the last session of the intervention (post-treatment).

##### Primary outcome

*General anxiety disorder 7 items (GAD-7; Spitzer et al., 2006; Ruiz et al., 2011).* This is a questionnaire consisting of 7 items to measure severity of GAD. Each item has a likert scale of four points (0 = “not at all”, 1 = “several days”, 2 = “more than half the days”, 3 = “nearly every day”). The total score (from 0 to 21) is divided into four categories of severity: without symptoms (0–4), mild anxiety symptoms (5–9), moderate anxiety symptoms (10–14) or severe anxiety symptoms (15–21). Therefore, higher scores mean more severity. The threshold score of 10 used for this measure has a sensitivity of 89% and a specificity of 82% for GAD (Kroenke et al., 2007). The scale has been validated in Spanish with patients attending primary health services and discriminates between patients with GAD and without GAD (Ruiz et al., 2011).

##### Secondary outcomes

*Hospital anxiety and depression scale (HADS; Zigmond and Snaith, 1983; Terol et al., 2007).* This scale evaluates the severity of anxiety and depression symptoms in non-psychiatric inpatients. It is composed of seven items that assess anxiety symptoms and seven for depression symptoms. Each item contains a scale of four points (from 0 to 3) with total scores ranging from 0 to 21 for anxiety and depression in three categorical levels: 0–7 = “normal”; 8–10 = “borderline abnormal”; 11–21 = “abnormal”. Higher scores mean greater severity. Psychometric properties for Spanish population showed an internal consistency and test-retest reliability equal or superior to 0.70 (Terol et al., 2007).

*Five facets of mindfulness questionnaire (FFMQ; Baer et al., 2006; Cebolla et al., 2012).* This questionnaire is composed of 39 items to evaluate five facets related with mindfulness. Each item is rated on a 5-point likert scale (1 = “never or very rarely true”; 5 = “very often or always true”). “Observing” is the ability to notice or attend to internal and external experiences such as sensations, thoughts, or emotions. “Describing” means to label internal experiences with words. “Acting with awareness” refers to focusing on one’s activities in the moment as opposed to behaving mechanically. “Non-judging of inner experience” means to take a non-evaluative stance toward thoughts and feelings and “non-reactivity to inner experience” refers to allowing thoughts and feelings to come and go, without getting caught up in or carried away by them (Cebolla et al., 2012). The Spanish version of this scale has shown good reliability and internal consistency (Cronbach’s alpha between 0.80 and 0.91; Cebolla et al., 2012).

*Difficulties of emotion regulation scale (DERS; Gratz and Roemer, 2004; Hervás and Jódar, 2008).* This scale (originally composed of 36 items) assesses areas of the emotion regulation process in which people can have difficulties. The Spanish validated version consists of 28 items that can be grouped into five subscales: inattention (“lack of emotional awareness, as a tendency not to attend to and acknowledge emotions”); confusion (“lack of emotional clarity or the extent to which individuals do not know –and are not clear about– the emotions they are experiencing”); non-acceptance (“tendency to have negative secondary emotional responses to one’s negative emotions, or non-accepting reactions to one’s distress”); interference (“difficulties concentrating and accomplishing tasks when experiencing negative emotion”)*;* and impulse (“difficulties remaining in control of one’s behavior when experiencing negative emotions”) (Gratz and Roemer, 2004). Participants score based on how often the items apply to themselves, using a 5-point Likert scale (1 = “almost never”; 5 = “almost always”). Higher scores mean greater difficulties with emotion regulation. The Spanish version of this scale has shown good psychometric properties (Cronbach’s alpha of 0.93 for the total score, and ranging from 0.78 to 0.93 for the subscales; Hervás and Jódar, 2008).

*Multidimensional assessment of interoceptive awareness (MAIA; Mehling et al., 2012).* It is a questionnaire composed of 32 items to measure the concept of interoceptive awareness on eight factors: (1) “Noticing” means being aware of uncomfortable, comfortable and neutral bodily sensations; (2) “Distracting” is defined as the tendency to ignore or distract oneself from sensations of pain or discomfort; (3) “Not-Worrying” is the tendency not to react with emotional distress or worry to sensations of pain or discomfort; (4) “Attention Regulation” is described as the ability to sustain and control attention to bodily sensation; (5) “Emotional Awareness” means being aware of the connection between bodily sensations and emotional states; (6) “Self-Regulation” is the ability to regulate psychological distress by attention to bodily sensations; (7) “Body Listening” means actively listening to the body for insight; and (8) “Trusting” means experiencing one’s body as safe and trustworthy (Mehling et al., 2012). Items are scored based on a 6-point Likert scale (0 = “never”; 5 = “always”). This questionnaire has shown good psychometric properties (Cronbach’s alpha: 0.66 to 0.87) for the original version (Mehling et al., 2012), and also good indicators in its preliminary Spanish version (Valenzuela-Moguillansky and Reyes-Reyes, 2015).

##### Virtual reality exploratory assessments

*Experience in the use of technologies.* A brief version of the Independent Television Company SOP Inventory, (ITC-SOPI; Lessiter et al., 2001; Baños et al., 2004) was used to assess previous experience with technologies at baseline in the MBI-VR group. Four questions were used to describe: (1) Experience with computers (answers choices were: “none”, “basic”, “intermediate”, and “expert”); (2) Knowledge about 3-D images (“none”, “basic”, “intermediate”, and “expert”); (3) Frequency of playing videogames (“never”, “occasionally: once or twice a month”, “frequently but less than 50% of the days”, “50% of the days or more”); (4) Knowledge about VR (“none”, “basic”, “intermediate”, and “expert”). Preliminary analyses of previous studies have demonstrated adequate psychometrics for the ITC-SOPI (Lessiter et al., 2001). This scale was administered at pre-treatment.

*Emotional state.* We used a visual analog scale with different emotions (VAS; Gross and Levenson, 1995) to assess the intensity of different emotions before and after VR. To decrease time burden on the volunteer participants, a briefer (seven item) version of the original measure (16 item) was used. Participants rate how they felt at that moment for the following emotions: “happiness”, “sadness”, “anger”, “surprise”, “anxiety”, “relax/calm”, and “vigor/energy” on a 7-point Likert scale (1 = “not feeling the emotion at all”; 7 = “feeling the emotion extremely”). This briefer 7-item scale has been used to evaluate emotional state in several studies (e.g., Riva et al., 2007). This scale was completed before and after each VR DBT^®^ mindfulness session.

*Sense of presence*. The Sense of Presence questionnaire was composed of three items with a 7-point Likert scale ranging from 1 to 7. The three items were adapted from the Slater, Usoh and Steed questionnaire (Slater et al., 1994), changing Slater et al.’s wording somewhat during the translation from English to Spanish (Navarro-Haro et al., 2017). Participants were asked to rate these three questions after each VR session: (1) “rate your sense of being in the virtual environment” (1 = “not at all”, 7 = “very much”); (2) “to what extent were there times during the experience when the virtual environment was reality for you?” (1 = “at no time”, 7 = “almost all of the time”); (3) “when you think back to the experience, do you think of the virtual environment more as images that you saw or more as some place that you visited?” (1 = “something I saw”, 7 = “some place I visited”). To simplify the analyses, these three items were summed as a presence component, with an internal consistence value in the present study of α = 0.92. The Spanish adaptation of this scale has been used to assess user’s presence while in virtual reality (e.g., Garcia-Palacios et al., 2002; Riva et al., 2007). This scale was rated after each VR DBT^®^ mindfulness session.

### Compliance With Ethical Standards

#### Ethics Statement

All procedures performed in studies involving human participants were in accordance with the ethical standards of the institutional and/or national research committee and with the 1964 Helsinki declaration and its later amendments or comparable ethical standards. This study was approved by the Clinical Research Ethics Committee of Aragón (Comité Ético de Investigación Clínica de Aragón, CEICA, reference code PI15/0050) which belongs to the Health Research Institute of Aragon (Instituto de Investigación Sanitaria de Aragón, IIS), from Zaragoza (Spain). Informed consent: Informed consent was obtained both verbally and written by all individual participants included in the present study.

### Statistical Analyses

The baseline characteristics of participants were described using means (*SD*) for the continuous variables, and frequencies (percentages) for the categorical variables. Baseline characteristics and group membership of participants were also evaluated according to the completion of the mindfulness program; those who attended ≥50% of mindfulness sessions were considered “completers” (Kuyken et al., 2008). The sub-group characteristics of participants were compared using the corresponding χ^2^ (or Fisher test when necessary) and *t*-tests.

The primary analysis was carried out for the primary outcome of GAD-7 in each group independently (MBI and MBI + VR DBT^®^) by using repeated measures mixed-effects linear regression by the restricted maximum likelihood (REML) method, in which time acts as an independent variable and the random part is assigned to subjects. REML is less biased for the estimation of variance parameters when using small sample sizes or unbalanced data (Egbewale et al., 2014). Unstandardized regression coefficients (*B*) and their 95% confidence interval (95% CI) were estimated (adjusting for the MAIA-body listening variable, which showed significant differences between groups at baseline). The effect size (ES) was reported by means of Cohen’s *d*, using the algorithm of Morris and DeShon (2002) for repeated measures, which is small when ≤0.20, medium when = 0.50, and large when ≥0.80.

Secondary analyses comprised comparisons of HADS, FFMQ, DERS, and MAIA, using the same analytical strategy described in the primary analyses. Moreover, we explored possible differences in the emotional state of the MBI+VR DBT^®^ group through each VR session, using the corresponding *t*-test for repeated measures, and the sense of presence after each VR session, using the above-mentioned repeated measures mixed-effects linear regression model by the REML method.

The overall α level was set at 0.05, using two-sided tests. Bonferroni’s adjustment for multiple comparisons was followed in the primary analyses, but secondary and exploratory analyses were considered as tentative, and therefore, we did not use corrections for multiple measurements (Feise, 2002). All analyses were performed using the STATA v12 package.

## Results

### Descriptive Statistics and Adherence to the Program

Table 1 shows descriptive statistics of the study variables at baseline for the total sample and by group. The levels of GAD-7 for the total group and by group were those corresponding to moderate anxiety symptoms (Total: *Mn* = 14.44; *SD* = 4.24; MBI: *Mn* = 14.80; *SD* = 3.94; MBI + VR: *Mn* = 14.05; *SD* = 4.61; *p* = 0.589). There were no significant differences at baseline for any of the socio-demographic or psychological outcomes by group, except for the body listening subscale of the MAIA (Total: *Mn* = 2.15; *SD* = 1.43; MBI: *Mn* = 1.67; *SD* = 1.37; MBI + VR: *Mn* = 2.69; *SD* = 1.33; *p* = 0.026), so that this variable was controlled in subsequent analyses. At post-treatment, there were *n* = 6 participants completing <50% of mindfulness sessions, and therefore, they were considered non-completers. As can be seen in Table 2, the MBI condition retained significantly fewer participants than the MBI + VR [MBI = 14 (70.0%); MBI + VR = 19 (100%); *Fisher* = 0.020]. Completion was also significantly associated with lower DERS-interference at baseline (completers: *Mn* = 13.16; *SD* = 3.39; non-completers: *Mn* = 17.20; *SD* = 1.79; *p* = 0.014). Having lower MAIA-not-worrying (*p* = 0.050), and higher MAIA-body-listening (*p* = 0.051), were not significantly related to completion but were in the predicted direction. Primary outcome data at *post*-test were obtained for 30 participants (71.4%).

**Table 2:** Baseline characteristics of participants by completion.

Variables	Completers (*n* = 33)	No-completers (*n* = 6)	*p*
*Group*			
MBI + VR	19 (100)	0 (0.0)	0.020
MBI	14 (70.0)	6 (30.0)	
*Socio-demographic*			
Age, mean (SD)	44.27 (10.25)	50.50 (15.68)	0.216
Gender, *n* (%)			
Female	26 (78.8)	4 (66.7)	0.607
Male	7 (21.2)	2 (33.3)	
Married/partner, *n* (%)			
Yes	18 (54.5)	4 (66.7)	0.679
No	15 (45.5)	2 (33.3)	
Studies, n (%)			
Primary/secondary	21 (63.7)	3 (50.0)	0.658
University	12 (36.3)	3 (50.0)	
Employment, *n* (%)			
Employed	17 (51.5)	2 (33.3)	0.661
No-employed^∗^	16 (48.5)	4 (66.7)	
Medication, *n* (%)			
Yes	22 (66.7)	4 (66.7)	1.000
No	11 (33.3)	2 (33.3)	
*Psychological*			
GAD-7, mean (SD)	14.33 (4.33)	15.00 (4.05)	0.728
HADS, mean (SD)			
Anxiety	13.09 (3.36)	14.50 (1.87)	0.327
Depression	8.91 (3.35)	11.33 (2.07)	0.097
FFMQ, mean (SD)			
Observing	26.79 (5.06)	23.00 (5.40)	0.103
Describing	25.21 (6.45)	22.00 (5.33)	0.259
Awareness	20.10 (4.83)	22.60 (5.55)	0.299
Non-judging	15.48 (6.06)	19.67 (9.85)	0.185
Non-reactivity	19.15 (3.99)	21.50 (3.45)	0.355
DERS, mean (SD)			
Inattention	10.52 (3.92)	12.00 (4.47)	0.446
Confusion	9.16 (2.60)	13.20 (5.89)	0.201
Non-acceptance	21.21 (8.15)	25.00 (9.25)	0.352
Interference	13.16 (3.39)	17.20 (1.79)	0.014
Impulse	24.76 (8.00)	23.80 (9.83)	0.812
MAIA, mean (SD)			
Noticing	4.05 (0.53)	3.67 (0.49)	0.109
Distracting	2.00 (0.97)	2.00 (1.28)	0.999
Not-worrying	1.81 (1.00)	2.78 (1.41)	0.050
Attention-regulation	2.92 (1.03)	2.00 (1.25)	0.062
Emotional-awareness	4.47 (1.49)	4.07 (0.59)	0.523
Self-regulation	2.39 (1.09)	1.79 (0.68)	0.205
Body-listening	2.34 (1.41)	1.11 (1.09)	0.051
Trusting	2.57 (1.54)	2.00 (1.49)	0.406


*MBI = mindfulness-based intervention; MBI + VR = mindfulness-based intervention + virtual reality; ^∗^stratum grouped including sick leave/retired; *p* = *p*-value as a result of χ^*2*^ (or Fisher) test for categorical variables, and *t*-test for continuous variables. *t*-test.*

### Primary and Secondary Outcome Pre-post Analyses

Primary and secondary analyses were performed based on completers. As can be seen in Table 3, there were significant pre-post improvements in GAD-7 for both the MBI (pre: *Mn* = 15.33; *SD* = 4.03; post: *Mn* = 9.08; *SD* = 3.85; *B* = -5.70; *p* < 0.001; *d* = -1.36), and the MBI + VR (pre: *Mn* = 14.05; *SD* = 4.61; post: *Mn* = 9.79; *SD* = 5.60; *B* = -4.38; *p* < 0.001; *d* = -1.33) groups taken separately. Moreover, as can be seen in Table 4, both sub-groups also reached significant pre-post improvements in HADS-anxiety, HADS-depression, FFMQ-describing, FFMQ-acting with awareness, DERS-confusion, DERS-impulse, MAIA-self-regulation, MAIA-body-listening, and MAIA-trusting. The MBI group exhibited additional improvements in DERS-inattention (*B* = -1.62; *p =* 0.016; *d* = -0.67); DERS-non-acceptance (*B* = -3.95; *p =* 0.011; *d* = -0.76); and MAIA-attention (*B* = 1.17; *p <* 0.001; *d* = 0.72). On the other hand, the MBI+VR group showed additional improvements in FFMQ-non-judging (*B* = 3.50; *p =* 0.024; *d* = 0.55); and DERS-interference (*B* = –2.39; *p <* 0.001; *d* = -0.84).

**Table 3:** Descriptive according to the group and primary and secondary outcome analyses.

	MBI + VR (*n* = 18)					MBI (*n* = 12)				
	Pre-	Post-	*B* (95% CI)	*p*	*d*	Pre-	Post-	*B* (95% CI)	*p*	*d*
*Primary outcome*										
GAD-7	14.05 (4.61)	9.79 (5.60)	–4.38 (–6.10 to –2.67)	<0.001	–1.33	15.33 (4.03)	9.08 (3.85)	–5.70 (–8.04 to –3.35)	< 0.001	–1.36
*Secondary outcomes*										
HADS anxiety	13.05 (4.10)	10.16 (4.72)	–2.89 (–4.45 to –1.33)	< 0.001	–0.96	13.33 (2.06)	10.08 (2.71)	–3.38 (–4.93 to –1.83)	< 0.001	–1.27
HADS depression	8.56 (3.79)	6.50 (3.97)	–2.06 (–3.87 to –0.24)	0.027	–0.54	9.33 (1.83)	5.50 (2.78)	–4.35 (–6.37 to –2.33)	< 0.001	–1.33
FFMQ observing	28.00 (4.77)	29.39 (4.43)	1.39 (–0.95 to 3.72)	0.244	0.26	24.75 (4.96)	26.83 (4.49)	2.29 (–0.69 to 5.27)	0.133	0.34
FFMQ describing	26.56 (4.85)	29.06 (5.29)	2.50 (0.97 to 4.03)	0.001	0.85	24.00 (8.57)	25.67 (9.22)	1.69 (0.31 to 3.07)	0.016	0.73
FFMQ awareness	20.12 (5.64)	23.29 (4.77)	3.18 (1.10 to 5.25)	0.003	0.66	20.00 (3.89)	23.42 (3.32)	3.14 (1.44 to 4.84)	< 0.001	1.07
FFMQ non-judging	16.56 (6.95)	20.06 (7.32)	3.50 (0.46 to 6.54)	0.024	0.55	13.67 (4.92)	17.75 (6.48)	2.66 (–1.61 to 6.92)	0.222	0.65
FFMQ non-reactivity	19.94 (3.84)	22.56 (8.33)	2.61 (–0.96 to 6.18)	0.152	0.58	17.42 (3.75)	18.83 (3.13)	0.83 (–1.04 to 2.71)	0.383	0.39
DERS inattention	9.56 (3.96)	8.67 (2.64)	–0.89 (–2.17 to 0.39)	0.172	–0.30	11.18 (3.16)	9.55 (2.16)	–1.62 (–2.93 to –0.30)	0.016	–0.67
DERS confusion	8.94 (2.51)	7.56 (2.57)	–1.39 (–2.50 to –0.28)	0.014	–0.59	8.18 (2.96)	7.91 (2.84)	–1.50 (–2.98 to –0.03)	0.045	–0.11
DERS non-acceptance	19.82 (8.55)	17.59 (6.69)	–2.37 (–6.01 to 1.27)	0.202	–0.28	23.20 (7.08)	19.20 (6.71)	–3.95 (–7.02 to –0.89)	0.011	–0.76
DERS interference	13.06 (3.51)	10.67 (2.99)	–2.39 (–3.65 to –1.13)	< 0.001	–0.84	13.45 (3.30)	12.18 (3.74)	–2.08 (–4.48 to 0.32)	0.090	–0.31
DERS impulse	22.53 (7.35)	18.24 (5.60)	–4.16 (–6.55 to –1.78)	0.001	–0.89	27.70 (8.11)	19.80 (4.34)	–6.55 (–10.98 to –2.13)	0.004	–0.88
MAIA noticing	4.15 (0.39)	4.25 (0.45)	0.11 (–0.16 to 0.38)	0.419	0.29	3.94 (0.63)	4.25 (0.49)	0.43 (–0.05 to 0.90)	0.080	0.37
MAIA distracting	2.15 (0.85)	1.91 (0.98)	–0.06 (–0.49 to 0.38)	0.802	–0.31	1.81 (1.14)	1.78 (0.86)	–0.05 (–0.79 to 0.69)	0.892	–0.02
MAIA non-worrying	1.80 (1.02)	1.94 (0.72)	0.11 (–0.24 to 0.46)	0.534	0.21	2.00 (1.00)	2.31 (0.89)	0.36 (–0.22 to 0.94)	0.220	0.28
MAIA attention	3.06 (1.03)	3.43 (0.76)	0.41 (–0.15 to 0.97)	0.150	0.29	2.74 (0.94)	3.54 (0.46)	1.17 (0.63 to 1.70)	< 0.001	0.72
MAIA emotional	4.34 (0.59)	4.43 (0.40)	0.11 (–0.24 to 0.46)	0.534	0.12	4.78 (2.30)	4.23 (0.63)	–0.31 (–1.43 to 0.80)	0.582	–0.17
MAIA self-regulation	2.49 (1.12)	3.57 (1.00)	1.11 (0.61 to 1.61)	< 0.001	0.98	2.33 (1.16)	3.60 (0.79)	1.34 (0.70 to 1.99)	< 0.001	0.89
MAIA body-listening	2.69 (1.33)	3.33 (0.86)	0.50 (0.09 to 0.91)	0.016	0.54	1.86 (1.36)	3.36 (0.67)	1.48 (0.97 to 2.00)	< 0.001	0.92
MAIA trusting	2.91 (1.17)	3.74 (0.88)	0.83 (0.26 to 1.41)	0.005	0.63	1.97 (1.81)	3.56 (1.16)	1.39 (0.50 to 2.29)	0.002	0.70


*Pre- and Post-treatment values are means (SD); *B* = unstandardized mixed effect regression coefficient. *p* = *p*-value. *d* = Cohen’s *d* effect size measure. Analyses controlling MAIA body-listening basal scores.*

**Table 4:** Descriptive and pre- post-session analyses of the emotional state (MBI + VR group).

		Session 1 (*n* = 19)			Session 2 (*n* = 18)			Session 3 (*n* = 19)			Session 4 (*n* = 19)			Session 5 (*n* = 18)			Session 6 (*n* = 17)		
		*Mn*	*SD*	*p*	*Mn*	*SD*	*p*	*Mn*	*SD*	*p*	*Mn*	*SD*	*p*	*Mn*	*SD*	*p*	*Mn*	*SD*	*p*
*Happiness*	pre-	3.84	1.46	0.083	3.94	0.90	0.248	3.68	1.06	0.160	3.89	1.10	0.097	3.78	1.31	0.361	4.12	1.17	0.248
	post-	4.16	1.54		4.18	1.19		3.95	0.97		4.26	1.33		4.00	1.37		4.35	1.41	
*Sadness*	pre-	3.37	1.80	0.018	3.00	1.03	0.001	3.37	1.42	0.046	2.95	1.13	0.032	3.17	1.20	0.046	2.71	0.85	0.265
	post-	2.68	1.45		2.17	0.86		2.89	1.10		2.26	0.99		2.72	1.41		2.41	1.00	
*Anger*	pre-	2.95	1.51	0.025	2.39	1.14	0.091	3.26	1.79	0.033	2.68	1.63	0.164	2.72	1.41	0.041	2.18	1.13	0.212
	post-	2.00	1.25		1.89	1.13		2.42	1.35		2.21	1.44		2.11	1.28		1.88	1.32	
*Surprise*	pre-	3.53	1.71	0.009	2.61	1.20	0.086	2.89	1.37	0.399	3.42	1.64	0.285	2.17	1.20	0.040	2.76	1.64	0.714
	post-	4.79	1.08		3.22	1.44		3.16	1.21		3.21	1.62		2.83	1.72		2.88	1.50	
*Anxiety*	pre-	4.58	1.30	0.002	4.17	1.10	0.005	4.16	1.34	0.067	3.84	1.38	0.065	3.94	1.30	0.001	3.53	1.12	0.049
	post-	3.21	1.72		3.11	1.49		3.53	1.43		3.16	1.50		3.00	1.41		2.88	1.45	
*Relaxation*	pre-	2.79	1.13	0.001	3.56	1.20	0.001	3.05	1.22	0.022	3.11	1.29	0.003	3.44	1.54	0.006	3.82	1.33	0.015
	post-	4.42	1.43		4.94	1.51		4.11	1.49		4.89	1.59		4.78	1.31		4.71	1.65	
*Vigor*	pre-	4.00	1.11	0.166	4.06	1.21	0.295	3.79	1.23	0.763	4.37	0.96	0.999	3.78	1.44	0.033	4.12	1.17	0.248
	post-	4.26	1.15		4.39	1.09		3.74	1.10		4.32	1.25		4.39	1.29		4.35	1.06	


*Pre- and Post-session values are means (Mn) and standard deviations (SD); *p* = *p*-value as a result of t-test.*

### Exploratory Results for the VR DBT^®^ Mindfulness Skills Training Sub-Group Regarding VR

Results showed that roughly half of participants of the MBI + VR DBT^®^ group at baseline had intermediate experience with computers (58.1%), 18.8% expert level and 23.3% basic level. Most participants had basic knowledge (27.9%) or no knowledge (60.5%) about 3-D images, and similar results were found for the knowledge about VR (27.9% basic, and 65.1% none). Most of them never played videogames (65.1%), and 30.2% played videogames occasionally.

Table 4 shows pre-post comparisons for each independent VR session on emotional state. Overall, these exploratory results indicate there were significant pre-post improvements in state of relaxation in all the VR sessions. Similar results were found for anxiety, although changes were non-significant in session number three (*p =* 0.067) and session number four (*p* = 0.065). The reductions of sadness were significant in all the sessions except for the last session (*p* = 0.265), in which the basal or starting levels were low compared with the other sessions. The state of anger was significantly reduced in three sessions; surprise was significantly increased in two sessions, and vigor was significantly increased in only one session. Finally, there were no significant changes in happiness in any of the VR sessions.

Table 5 presents the evolution of the post-session sense of presence values along the treatment with VR. Results pointed to initial decrements in presence with a significant change of tendency in the middle of the intervention, from which increases were observed. Specifically, presence had gone down in the first half of the program (session 1 vs. session 3: *B* = -1.87; *p* = 0.022), but it returned to baseline levels at the final of the program [(session 3 vs. session 6: *B* = 2.13; *p* = 0.002), (session 1 vs. session 6: *B* = 0.27; *p* = 0.744)].

**Table 5:** Descriptive and post-session comparisons on sense of presence (MBI + VR group).

			Comparisons^∗^					
		*Mn (SD)*	*vs. 1*	*vs. 2*	*vs. 3*	*vs. 4*	*vs. 5*	*vs. 6*
SUS (*n* = 15)	*Session 1*	14.80 (4.16)	–	–0.87 (*p* = 0.289)	–1.87 (*p* = 0.022)	–0.80 (*p* = 0.327)	–0.33 (*p* = 0.683)	0.27 (*p* = 0.744)
	*Session 2*	13.93 (4.59)		–	–1.00 (*p* = 0.203)	0.07 (*p* = 0.932)	0.53 (*p* = 0.497)	1.13 (*p* = 0.149)
	*Session 3*	12.93 (5.33)			–	1.07 (*p* = 0.125)	1.53 (*p* = 0.028)	2.13 (*p* = 0.002)
	*Session 4*	14.00 (5.26)				–	0.47 (*p* = 0.421)	1.07 (*p* = 0.066)
	*Session 5*	14.47 (4.41)					–	1.07 (*p* = 0.074)
	*Session 6*	15.07 (4.85)						–


*^∗^Values are *B* coefficients using mixed-effect regression models (*p*-values in brackets).*

## Discussion

At the time of writing, this is the first study to explore the use of VR DBT^®^ mindfulness skills training sessions to enhance a mindfulness-based intervention for the treatment of patients with GAD in PC. In addition, although it has been recommended in several reviews, there have been very few studies investigating MBIs to treat GAD in general.

The primary hypothesis of this study was that participants receiving the MBI, with or without the addition of VR, would show significant decreases in GAD symptoms. Results confirm this hypothesis since there were statistically significant decreases, with large effect sizes in the primary outcome (GAD-7) for both MBI and MBI+VR DBT^®^ groups. Although other mindfulness-based approaches had tested a MBI for treating some symptoms of GAD (Craigie et al., 2008; Evans et al., 2008), the current study is the first study that evaluates the effect of a MBI to improve GAD symptoms all together –i.e., it evaluates a larger number of GAD related symptoms than the referred previous studies. In general, the current results add evidence to the literature for using an MBI to treat GAD symptoms.

As secondary hypotheses, we expected that both MBI and MBI+VR DBT^®^ groups would be efficacious for the improvement of other variables associated with GAD. Results showed significant pre-post improvements and moderate to large effect sizes for both groups in a large part of the secondary outcomes, including statistically significant improvements in anxiety and depression symptoms that are in the line with previous studies (Craigie et al., 2008; Hoge et al., 2013). In addition to replicating the findings of Craigie et al. (2008) and Hoge et al. (2013), this study also found statistically significant improvements in some of the difficulties of emotion regulation factors, such as confusion and impulse. Emotion regulation has been proposed as a transdiagnostic factor influencing vulnerability to different emotional disorders (Campbell-Sills and Barlow, 2007). Given the high comorbidity between GAD and other emotional disorders, these results suggest that the MBI used might help to treat the associated emotional disorders. Furthermore, there were significant improvements in aspects related to mindfulness and awareness. Specifically, results of both groups indicate significant increases in facets of mindfulness, such as describing and acting with awareness, and several aspects of interoceptive awareness, such as self-regulation, body-listening, and trusting. This is the first study showing preliminary positive outcomes of different aspects of mindfulness and interoceptive awareness after a brief MBI intervention for GAD. In general, these findings are very encouraging. By increasing mindfulness and awareness, the MBI might help to decrease rumination (Raes and Williams, 2010) and may reduce difficulties in awareness of internal experiences (Hayes and Feldman, 2004) common in GAD patients.

The MBI+VR DBT^®^ mindfulness skills training group showed additional pre-post improvements in the non-judging facet of mindfulness and the interference subscale – defined as difficulties concentrating and accomplishing tasks when experiencing negative emotion as measured by the Difficulties of Emotion Regulation Scale **(**DERS). It might be that practicing DBT^®^ mindfulness exercises through VR help enhance important aspects of self-regulation when experiencing negative emotions (Navarro-Haro et al., 2016). On the other hand, the MBI group exhibited additional pre-post improvements in two types of difficulties of emotion regulation (inattention and non-acceptance) and increases in the attention aspects of interoceptive awareness. Overall, these results from both groups suggest that this type of intervention (i.e., MBI) might be specifically effective for addressing attention to the experience in GAD patients.

We also expected that the group receiving VR DBT^®^ mindfulness skills would be more adherent to the intervention than the MBI alone. Results confirmed our hypothesis given that the MBI+VR condition retained significantly more participants than the MBI alone. This outcome is very encouraging since the main goal of using VR was to enhance the MBI. We believe this can be a very important research direction since the previously reported rates of drop-outs for GAD patients are high. Therefore, VR DBT^®^ mindfulness might become a good tool to increase treatment adherence and motivation to practice mindfulness when delivering mindfulness-based approaches (Navarro-Haro et al., 2017). The current is the first study to use VR DBT^®^ mindfulness skills to help treating GAD. Future research should explore whether using VR DBT^®^ mindfulness skills during one-on-one therapy sessions could be unusually effective for treating GAD.

Additionally, we explored other predictors of treatment adherence. This is a very new research line since we only found one review of predictors to treatment adherence for anxiety disorders and it did not include GAD patients (Santana and Fontenelle, 2011). The authors of the review did not find consistent conclusions regarding general predictors of treatment adherence and emphasized the importance of more research on this topic. In the current study, completion was significantly associated with lower scores in the interference scale of the DERS scale at baseline. On the other hand, having lower scores in not-worrying, and higher scores in body-listening (aspects of interoceptive awareness), although they were not significantly related to completion, these results were in the predicted direction. Working on difficulties to regulate emotions, worry thoughts and body listening might be good targets to be included early in the future development of treatments for GAD.

Finally, we wanted to explore the effect of each VR session separately. We expected that participants would at least temporarily improve their emotional state immediately after each of the VR sessions. Results partially confirmed our hypothesis since only the state of relaxation was improved in all the sessions. Nonetheless, given that GAD patients have difficulties changing their mood, the findings that patients reported short-term reductions in negative emotions after the VR DBT^®^ Mindfulness Skills training is a very encouraging outcome and goes in the line with previous case series studies with other clinical patient populations (Gomez et al., 2017; Navarro-Haro et al., 2016; Flores et al., 2018). Regarding sense of presence, results showed a significant change of tendency from the middle of the intervention, suggesting that patients were able to experience the illusion of going inside the VR, as if it is a place they were visiting at treatment. In a previous study using the same VR system with mindfulness experts (Navarro-Haro et al., 2017), sense of presence significantly correlated with acceptability. Although we did not include the sense of presence as a predictor of treatment adherence (because it was only used in the MBI + VR DBT^®^ condition), this result may point out a key role that sense of presence plays in the users’ acceptance using VR to practice mindfulness.

This pilot study has several important limitations. Firstly, pre-post studies are scientifically inconclusive by nature, and the results should be interpreted with caution. Secondly, the sample sizes used were too small to analyse with enough statistical power possible direct comparisons between both the MBI and MBI + VR DBT^®^ groups, and therefore, more powerful studies using between-group comparisons and including active and passive control groups are needed to reach higher levels of evidence to test the hypotheses. Alternatively, a within-subjects design could administer measures after the main treatment session, and again after the VR session, to see if the VR session further reduced the patient’s symptoms. In addition, the link between data and conclusions regarding the effect of each VR session separately is not strong because there were no comparisons for emotional states for each session in other group functioning as a control. Thus, it is difficult to isolate the specific factors that cause changes beyond the simple attention received. In this sense, the novelty of using VR might be a factor influencing results in the MBI+VR group. Another limitation is the lack of follow-up measures of long-term outcome (e.g., 6 month or 12 month after completing treatment). Additionally, in the VR-enhanced treatment group, the primary treatment (the MBI) did not involve virtual reality during the group sessions. Virtual reality was only used during 10 min before or after their 50-min traditional MBI session. Thus, future studies that more fully implement VR enhancement of MBI therapy sessions are needed. These future studies that also use VR enhanced mindfulness during the primary treatment sessions, should provide a more statistically powerful test of VR enhanced MBI treatment.

Despite these limitations, the current study yielded a number of interesting findings. Excessive worrying can highly interference with everyday life, and is a very prevalent problem in GAD. It is associated to high comorbidity with mood and anxiety disorders and shows low levels of mindfulness and emotion regulation (Tyrer and Baldwin, 2006; Roemer et al., 2009). Results of the present study has shown that both MBI and MBI + VR DBT^®^ separately reached significant gains in GAD symptoms, anxiety, depression and some aspects of difficulties of emotion regulation, mindfulness and interoceptive awareness after treatment. VR was used in this study as a possible tool to enhance the MBI intervention. VR combined with MBI retained significantly more participants in the treatment than those that received only MBI. Using VR sessions to practice mindfulness seemed to increase state of relaxation in all the sessions and the current study observed changes in presence over time. Overall VR continued to yield a strong illusion of presence, even when used repeatedly.

Previous studies have shown that anxious patients are much more willing to seek treatment delivered via therapists plus virtual reality (e.g., virtual spiders), compared to traditional therapy with no virtual reality – e.g., “*in vivo*” vs. virtual exposure therapy for spider phobia (García-Palacios et al., 2007). Similarly, patients with Post-traumatic Stress Disorder (PTSD) are much more likely to seek VR exposure therapy for PTSD compared to conventional exposure therapy for PTSD (Botella et al., 2015). Patients with GAD could be more likely to seek treatment when the program involves VR, and also using VR to enhance mindfulness-based interventions could increase treatment acceptance rates.

In general, this exploratory study adds preliminary effectiveness of a MBI to treat GAD symptoms, depression, anxiety, and emotion dysregulation to the previous literature. Furthermore, VR might become a good tool to increase treatment adherence and motivation to practice mindfulness when delivering mindfulness-based approaches for GAD. However, more studies using more statistically powerful designs that allow direct comparisons with specific and non-specific control groups are warranted.

## Author Contributions

MN-H coordinated the data collection, study design, and led the manuscript development process. MM-A assisted in data collection and contributed to the manuscript. MN-G assisted in data collection. AL-M assisted in the interventions implementation and contributed to the manuscript. LB coordinated the data collection and contributed to the manuscript. JM-M carried-out the analysis of data, and development and writing of the diverse drafts and manuscripts. HH contributed to the manuscript development process and study coordination. AG-P contributed to the manuscript development. JG-C oversaw the development, the implementation of the study, and contributed to the manuscript.

## Conflict of Interest Statement

The authors declare that the research was conducted in the absence of any commercial or financial relationships that could be construed as a potential conflict of interest.

## Footnotes

1 www.bigenvironment.com
